# *N*′-(2,6-Difluoro­benzyl­idene)pyridine-4-carbohydrazide

**DOI:** 10.1107/S1600536812016716

**Published:** 2012-04-21

**Authors:** Hoong-Kun Fun, Ching Kheng Quah, Divya N. Shetty, B. Narayana, B. K. Sarojini

**Affiliations:** a X-ray Crystallography Unit, School of Physics, Universiti Sains Malaysia, 11800 USM, Penang, Malaysia; b Department of Studies in Chemistry, Mangalore University, Mangalagangotri 574 199, India; c Department of Chemistry, P. A. College of Engineering, Nadupadavu, Mangalore 574 153, India

## Abstract

In the title compound, C_13_H_9_F_2_N_3_O, the pyridine ring forms a dihedral angle of 16.92 (7)° with the benzene ring. In the crystal, mol­ecules are linked *via* N—H⋯O, C—H⋯O and C—H⋯F, with the same O atom accepting two bonds.

## Related literature

For related structures, see: Chen (2006 ▶); Nie *et al.* (2006 ▶). For standard bond-length data, see: Allen *et al.* (1987 ▶). For the stability of the temperature controller used in the data collection, see: Cosier & Glazer (1986 ▶).
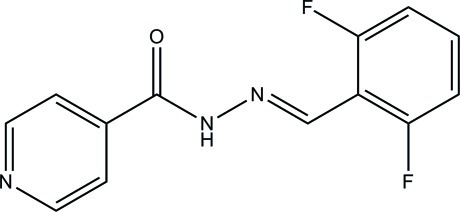


## Experimental

### 

#### Crystal data

C_13_H_9_F_2_N_3_O
*M* *_r_* = 261.23
Monoclinic, 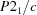

*a* = 6.8462 (1) Å
*b* = 24.7903 (5) Å
*c* = 8.3719 (1) Å
β = 125.249 (1)°
*V* = 1160.36 (3) Å^3^
*Z* = 4
Mo *K*α radiation
μ = 0.12 mm^−1^
*T* = 100 K
0.68 × 0.26 × 0.10 mm

#### Data collection

Bruker SMART APEXII CCD area-detector diffractometer
Absorption correction: multi-scan (*SADABS*; Bruker, 2009 ▶) *T*_min_ = 0.923, *T*_max_ = 0.988
22044 measured reflections
4531 independent reflections
3819 reflections with *I* > 2σ(*I*)
*R*_int_ = 0.029

#### Refinement

*R*[*F* ^2^ > 2σ(*F* ^2^)] = 0.045
*wR*(*F* ^2^) = 0.117
*S* = 1.06
4531 reflections
176 parameters
H atoms treated by a mixture of independent and constrained refinement
Δρ_max_ = 0.50 e Å^−3^
Δρ_min_ = −0.23 e Å^−3^

### 

Data collection: *APEX2* (Bruker, 2009 ▶); cell refinement: *SAINT* (Bruker, 2009 ▶); data reduction: *SAINT*; program(s) used to solve structure: *SHELXTL* (Sheldrick, 2008 ▶); program(s) used to refine structure: *SHELXTL*; molecular graphics: *SHELXTL*; software used to prepare material for publication: *SHELXTL* and *PLATON* (Spek, 2009 ▶).

## Supplementary Material

Crystal structure: contains datablock(s) global, I. DOI: 10.1107/S1600536812016716/bv2204sup1.cif

Structure factors: contains datablock(s) I. DOI: 10.1107/S1600536812016716/bv2204Isup2.hkl

Supplementary material file. DOI: 10.1107/S1600536812016716/bv2204Isup3.cml

Additional supplementary materials:  crystallographic information; 3D view; checkCIF report

## Figures and Tables

**Table 1:** Hydrogen-bond geometry (Å, °)

*D*—H⋯*A*	*D*—H	H⋯*A*	*D*⋯*A*	*D*—H⋯*A*
N2—H1*N*2⋯O1^i^	0.88 (2)	2.00 (2)	2.8554 (12)	163.4 (16)
C1—H1*A*⋯F1^ii^	0.93	2.54	3.4622 (13)	169
C7—H7*A*⋯O1^i^	0.93	2.45	3.2253 (13)	141

Symmetry codes: (i) 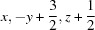; (ii) 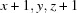.

**Fig. 1.**
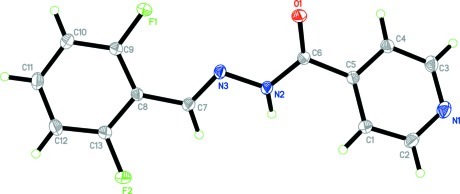
The molecular structure of the title compound showing 50% probability displacement ellipsoids for non-H atoms.

**Fig. 2.**
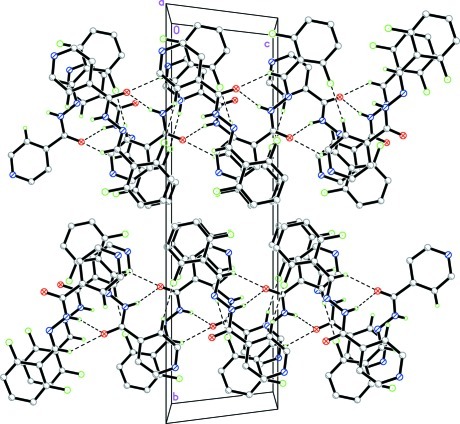
The crystal structure of the title compound, viewed along the a axis. H atoms not involved in hydrogen bonds (dashed lines) have been omitted for clarity.

## supplementary crystallographic information

### Comment

In view of the importance of isoniazid and its various Schiff base derivatives (Chen, 2006; Nie *et al.*, 2006), the synthesis and crystal structure of the title Schiff base is reported.

In the title molecule (Fig. 1), the pyridine ring (N1/C1-C5) forms a dihedral angle of 16.92 (7)° with the benzene ring (C8-C13). Bond lengths (Allen *et al.*, 1987) and angles are within normal ranges and are comparable with related structures (Chen, 2006; Nie *et al.*, 2006).

In the crystal structure, Fig. 2, molecules are linked *via* intermolecular C1–H1A···F1 and bifurcated N2–H1N2···O1 and C7–H7A···O1 hydrogen bonds (Table 1) into two-dimensional planes parallel to (010).

### Experimental

A mixture of isoniazid (1.4 g, 0.01 mol) and 2,6-difluorobenzaldehyde (1.4 ml, 0.01 mol) in 15 ml of absolute alcohol containing 2 drops of hydrochloric acid was refluxed for about 3 h. On cooling, the solid was separated, which was then filtered and recrystallized from DMF. The single crystal was grown from DMF by the slow evaporation method. (*m.p.* > 523 K).

### Refinement

Atom H1N2 was located in a difference Fourier map and refined freely with N2-H1N2 = 0.884 (18) Å. The remaining H atoms were positioned geometrically and refined using a riding model with C–H = 0.93 Å and *U*_iso_(H) = 1.2 *U*_eq_(C).

### Crystal data

**Table:**

C_13_H_9_F_2_N_3_O	*F*(000) = 536
*M**_r_* = 261.23	*D*_x_ = 1.495 Mg m^−^^3^
Monoclinic, *P*2_1_/*c*	Mo *K*α radiation, λ = 0.71073 Å
Hall symbol: -P 2ybc	Cell parameters from 8606 reflections
*a* = 6.8462 (1) Å	θ = 3.1–33.5°
*b* = 24.7903 (5) Å	µ = 0.12 mm^−^^1^
*c* = 8.3719 (1) Å	*T* = 100 K
β = 125.249 (1)°	Plate, colourless
*V* = 1160.36 (3) Å^3^	0.68 × 0.26 × 0.10 mm
*Z* = 4	

### Data collection

**Table:**

Bruker SMART APEXII CCD area-detector diffractometer	4531 independent reflections
Radiation source: fine-focus sealed tube	3819 reflections with *I* > 2σ(*I*)
Graphite monochromator	*R*_int_ = 0.029
φ and ω scans	θ_max_ = 33.5°, θ_min_ = 3.1°
Absorption correction: multi-scan (*SADABS*; Bruker, 2009)	*h* = −10→10
*T*_min_ = 0.923, *T*_max_ = 0.988	*k* = −38→38
22044 measured reflections	*l* = −12→12

### Refinement

**Table:**

Refinement on *F*^2^	Primary atom site location: structure-invariant direct methods
Least-squares matrix: full	Secondary atom site location: difference Fourier map
*R*[*F*^2^ > 2σ(*F*^2^)] = 0.045	Hydrogen site location: inferred from neighbouring sites
*wR*(*F*^2^) = 0.117	H atoms treated by a mixture of independent and constrained refinement
*S* = 1.06	*w* = 1/[σ^2^(*F*_o_^2^) + (0.0562*P*)^2^ + 0.3643*P*] where *P* = (*F*_o_^2^ + 2*F*_c_^2^)/3
4531 reflections	(Δ/σ)_max_ = 0.001
176 parameters	Δρ_max_ = 0.50 e Å^−^^3^
0 restraints	Δρ_min_ = −0.23 e Å^−^^3^

### Special details

**Table:**

Experimental. The crystal was placed in the cold stream of an Oxford Cryosystems Cobra open-flow nitrogen cryostat (Cosier & Glazer, 1986) operating at 100.0 (1) K.
Geometry. All esds (except the esd in the dihedral angle between two l.s. planes) are estimated using the full covariance matrix. The cell esds are taken into account individually in the estimation of esds in distances, angles and torsion angles; correlations between esds in cell parameters are only used when they are defined by crystal symmetry. An approximate (isotropic) treatment of cell esds is used for estimating esds involving l.s. planes.
Refinement. Refinement of F^2^ against ALL reflections. The weighted R-factor wR and goodness of fit S are based on F^2^, conventional R-factors R are based on F, with F set to zero for negative F^2^. The threshold expression of F^2^ > 2sigma(F^2^) is used only for calculating R-factors(gt) etc. and is not relevant to the choice of reflections for refinement. R-factors based on F^2^ are statistically about twice as large as those based on F, and R- factors based on ALL data will be even larger.

### Fractional atomic coordinates and isotropic or equivalent isotropic displacement parameters (Å^2^)

**Table:**

	*x*	*y*	*z*	*U*_iso_*/*U*_eq_	
F1	0.41487 (12)	0.82429 (3)	−0.46332 (9)	0.02284 (14)	
F2	0.74397 (12)	0.94647 (3)	0.06279 (9)	0.02428 (15)	
O1	0.88240 (14)	0.69293 (3)	−0.10748 (11)	0.01802 (15)	
N1	1.26302 (17)	0.60373 (4)	0.54592 (13)	0.02081 (18)	
N2	0.85867 (15)	0.75837 (3)	0.07264 (12)	0.01533 (15)	
N3	0.73916 (15)	0.79285 (3)	−0.08611 (12)	0.01499 (15)	
C1	1.20146 (17)	0.69340 (4)	0.41379 (14)	0.01558 (17)	
H1A	1.2365	0.7300	0.4362	0.019*	
C2	1.30695 (18)	0.65690 (4)	0.56804 (15)	0.01889 (18)	
H2A	1.4143	0.6701	0.6940	0.023*	
C3	1.11063 (19)	0.58554 (4)	0.36284 (16)	0.01903 (19)	
H3A	1.0787	0.5487	0.3448	0.023*	
C4	0.99779 (17)	0.61841 (4)	0.19868 (15)	0.01578 (17)	
H4A	0.8951	0.6039	0.0741	0.019*	
C5	1.04182 (16)	0.67368 (4)	0.22471 (13)	0.01332 (16)	
C6	0.92010 (16)	0.70905 (4)	0.04740 (14)	0.01366 (16)	
C7	0.71196 (16)	0.84069 (4)	−0.04226 (14)	0.01461 (16)	
H7A	0.7767	0.8489	0.0877	0.018*	
C8	0.58232 (16)	0.88247 (4)	−0.19069 (14)	0.01341 (16)	
C9	0.43387 (17)	0.87443 (4)	−0.39305 (14)	0.01554 (17)	
C10	0.30493 (19)	0.91512 (4)	−0.52600 (15)	0.01971 (19)	
H10A	0.2063	0.9079	−0.6594	0.024*	
C11	0.3256 (2)	0.96720 (4)	−0.45622 (16)	0.0218 (2)	
H11A	0.2382	0.9950	−0.5441	0.026*	
C12	0.47472 (19)	0.97845 (4)	−0.25729 (16)	0.02061 (19)	
H12A	0.4914	1.0134	−0.2107	0.025*	
C13	0.59696 (17)	0.93600 (4)	−0.13148 (14)	0.01623 (17)	
H1N2	0.870 (3)	0.7667 (7)	0.180 (3)	0.034 (4)*	

### Atomic displacement parameters (Å^2^)

**Table:**

	*U*^11^	*U*^22^	*U*^33^	*U*^12^	*U*^13^	*U*^23^
F1	0.0293 (3)	0.0157 (3)	0.0164 (3)	0.0010 (2)	0.0091 (3)	−0.0034 (2)
F2	0.0308 (3)	0.0188 (3)	0.0156 (3)	0.0007 (2)	0.0089 (3)	−0.0043 (2)
O1	0.0264 (4)	0.0163 (3)	0.0134 (3)	0.0014 (3)	0.0126 (3)	−0.0004 (2)
N1	0.0226 (4)	0.0217 (4)	0.0176 (4)	0.0037 (3)	0.0113 (3)	0.0052 (3)
N2	0.0211 (4)	0.0142 (3)	0.0117 (3)	0.0042 (3)	0.0100 (3)	0.0025 (3)
N3	0.0182 (3)	0.0143 (3)	0.0135 (3)	0.0027 (3)	0.0097 (3)	0.0029 (3)
C1	0.0165 (4)	0.0160 (4)	0.0139 (4)	0.0004 (3)	0.0086 (3)	−0.0003 (3)
C2	0.0183 (4)	0.0226 (4)	0.0133 (4)	0.0022 (3)	0.0077 (4)	0.0013 (3)
C3	0.0222 (4)	0.0154 (4)	0.0204 (5)	0.0021 (3)	0.0128 (4)	0.0037 (3)
C4	0.0175 (4)	0.0146 (4)	0.0158 (4)	0.0003 (3)	0.0100 (3)	0.0003 (3)
C5	0.0154 (4)	0.0135 (3)	0.0130 (4)	0.0014 (3)	0.0093 (3)	0.0011 (3)
C6	0.0154 (4)	0.0136 (3)	0.0127 (4)	0.0001 (3)	0.0085 (3)	0.0004 (3)
C7	0.0162 (4)	0.0149 (4)	0.0131 (4)	0.0010 (3)	0.0086 (3)	0.0009 (3)
C8	0.0155 (4)	0.0124 (3)	0.0138 (4)	0.0007 (3)	0.0093 (3)	0.0005 (3)
C9	0.0180 (4)	0.0139 (4)	0.0149 (4)	−0.0001 (3)	0.0096 (3)	−0.0009 (3)
C10	0.0225 (4)	0.0199 (4)	0.0143 (4)	0.0025 (3)	0.0092 (4)	0.0032 (3)
C11	0.0269 (5)	0.0168 (4)	0.0210 (5)	0.0048 (3)	0.0135 (4)	0.0061 (4)
C12	0.0266 (5)	0.0135 (4)	0.0224 (5)	0.0020 (3)	0.0146 (4)	0.0015 (3)
C13	0.0192 (4)	0.0146 (4)	0.0150 (4)	−0.0003 (3)	0.0099 (3)	−0.0012 (3)

### Geometric parameters (Å, º)

**Table:**

F1—C9	1.3488 (11)	C4—C5	1.3928 (13)
F2—C13	1.3550 (12)	C4—H4A	0.9300
O1—C6	1.2334 (11)	C5—C6	1.4963 (13)
N1—C3	1.3399 (14)	C7—C8	1.4601 (13)
N1—C2	1.3410 (14)	C7—H7A	0.9300
N2—C6	1.3485 (12)	C8—C9	1.3983 (13)
N2—N3	1.3826 (11)	C8—C13	1.3998 (13)
N2—H1N2	0.884 (18)	C9—C10	1.3790 (14)
N3—C7	1.2864 (12)	C10—C11	1.3901 (15)
C1—C2	1.3897 (14)	C10—H10A	0.9300
C1—C5	1.3936 (13)	C11—C12	1.3897 (16)
C1—H1A	0.9300	C11—H11A	0.9300
C2—H2A	0.9300	C12—C13	1.3783 (14)
C3—C4	1.3872 (14)	C12—H12A	0.9300
C3—H3A	0.9300		
			
C3—N1—C2	116.95 (9)	N2—C6—C5	114.91 (8)
C6—N2—N3	118.36 (8)	N3—C7—C8	121.88 (9)
C6—N2—H1N2	121.3 (11)	N3—C7—H7A	119.1
N3—N2—H1N2	119.4 (11)	C8—C7—H7A	119.1
C7—N3—N2	113.49 (8)	C9—C8—C13	114.68 (8)
C2—C1—C5	118.26 (9)	C9—C8—C7	126.14 (8)
C2—C1—H1A	120.9	C13—C8—C7	119.14 (9)
C5—C1—H1A	120.9	F1—C9—C10	117.80 (9)
N1—C2—C1	123.84 (9)	F1—C9—C8	118.65 (8)
N1—C2—H2A	118.1	C10—C9—C8	123.55 (9)
C1—C2—H2A	118.1	C9—C10—C11	118.53 (10)
N1—C3—C4	123.81 (9)	C9—C10—H10A	120.7
N1—C3—H3A	118.1	C11—C10—H10A	120.7
C4—C3—H3A	118.1	C12—C11—C10	121.09 (9)
C3—C4—C5	118.45 (9)	C12—C11—H11A	119.5
C3—C4—H4A	120.8	C10—C11—H11A	119.5
C5—C4—H4A	120.8	C13—C12—C11	117.68 (9)
C4—C5—C1	118.67 (9)	C13—C12—H12A	121.2
C4—C5—C6	118.35 (8)	C11—C12—H12A	121.2
C1—C5—C6	122.96 (8)	F2—C13—C12	118.28 (9)
O1—C6—N2	124.30 (9)	F2—C13—C8	117.29 (8)
O1—C6—C5	120.79 (8)	C12—C13—C8	124.43 (9)
			
C6—N2—N3—C7	−172.82 (9)	N3—C7—C8—C9	14.15 (15)
C3—N1—C2—C1	1.03 (16)	N3—C7—C8—C13	−167.93 (9)
C5—C1—C2—N1	−0.46 (15)	C13—C8—C9—F1	178.07 (8)
C2—N1—C3—C4	−0.28 (16)	C7—C8—C9—F1	−3.93 (15)
N1—C3—C4—C5	−0.99 (15)	C13—C8—C9—C10	−1.87 (14)
C3—C4—C5—C1	1.52 (14)	C7—C8—C9—C10	176.13 (10)
C3—C4—C5—C6	179.93 (9)	F1—C9—C10—C11	−178.93 (9)
C2—C1—C5—C4	−0.85 (14)	C8—C9—C10—C11	1.00 (16)
C2—C1—C5—C6	−179.18 (9)	C9—C10—C11—C12	0.75 (17)
N3—N2—C6—O1	2.02 (14)	C10—C11—C12—C13	−1.46 (17)
N3—N2—C6—C5	−178.14 (8)	C11—C12—C13—F2	179.64 (9)
C4—C5—C6—O1	−34.71 (13)	C11—C12—C13—C8	0.49 (16)
C1—C5—C6—O1	143.63 (10)	C9—C8—C13—F2	−178.05 (9)
C4—C5—C6—N2	145.45 (9)	C7—C8—C13—F2	3.79 (13)
C1—C5—C6—N2	−36.22 (13)	C9—C8—C13—C12	1.10 (15)
N2—N3—C7—C8	−177.64 (8)	C7—C8—C13—C12	−177.05 (9)

### Hydrogen-bond geometry (Å, º)

**Table:**

*D*—H···*A*	*D*—H	H···*A*	*D*···*A*	*D*—H···*A*
N2—H1*N*2···O1^i^	0.88 (2)	2.00 (2)	2.8554 (12)	163.4 (16)
C1—H1*A*···F1^ii^	0.93	2.54	3.4622 (13)	169
C7—H7*A*···O1^i^	0.93	2.45	3.2253 (13)	141

Symmetry codes: (i) *x*, −*y*+3/2, *z*+1/2; (ii) *x*+1, *y*, *z*+1.

## References

[bb1] Allen, F. H., Kennard, O., Watson, D. G., Brammer, L., Orpen, A. G. & Taylor, R. (1987). *J. Chem. Soc. Perkin Trans. 2*, pp. S1–19.

[bb2] Bruker (2009). *APEX2*, *SAINT* and *SADABS* Bruker AXS Inc., Madison, Wisconsin, USA.

[bb3] Chen, S.-K. (2006). *Acta Cryst.* E**62**, o5352–o5353.

[bb4] Cosier, J. & Glazer, A. M. (1986). *J. Appl. Cryst.* **19**, 105–107.

[bb5] Nie, A., Ghosh, S. & Huang, Z. (2006). *Acta Cryst.* E**62**, o1824–o1825.

[bb6] Sheldrick, G. M. (2008). *Acta Cryst.* A**64**, 112–122. 10.1107/S0108767307043930 18156677

[bb7] Spek, A. L. (2009). *Acta Cryst.* D**65**, 148–155. 10.1107/S090744490804362X PMC2631630 19171970

